# Arabic consonant length perception depends on the relative speech rate of the distal context

**DOI:** 10.1371/journal.pone.0312249

**Published:** 2024-10-22

**Authors:** Christopher C. Heffner, Buthainah M. Al-Thowaini, C. Anton Rytting

**Affiliations:** 1 Program in Neuroscience and Cognitive Science, University of Maryland, College Park, Maryland, United States of America; 2 Department of Linguistics, University of Maryland, College Park, Maryland, United States of America; 3 Department of Hearing and Speech Sciences, University of Maryland, College Park, Maryland, United States of America; 4 Department of English, College of Language Sciences, King Saud University, Riyadh, Kingdom of Saudi Arabia; 5 Center for Advanced Study of Language, University of Maryland, College Park, Maryland, United States of America; Cyprus University of Technology, CYPRUS

## Abstract

Listeners adapt to the speech rate of talkers. Many studies of speech rate adaptation have focused on the influence of rate information on the perception of word segmentation or segmental perception in English. The effects of immediately adjacent (i.e., proximal) information are generally strong on the perception of both segments and segmentation, but the effects of rate information temporally remote from (i.e., distal to) ambiguous speech signals are less clear, especially for segments. The present study examines the influence of distal rate adaptation on the perception of geminate consonants in Arabic that straddle a morpheme boundary (i.e., heteromorphemic geminates). Participants heard sentences that at one point were ambiguous to the presence of the Arabic definite clitic *al*, which, under certain circumstances, can be realized as gemination of the subsequent word-initial consonant. The sentences were either recorded with or without the clitic and with three possible distal speech rates in the context of the clitic. They transcribed the sentences and reported what they heard, and those transcriptions were analyzed for the contribution of the original recording and the distal speech rate on the perception of *al*. It was found that the perception of geminates in Arabic is rate dependent. This extends the knowledge of the effects of distal rate cues to Arabic, showing that Arabic geminate consonants are perceived relative to the rate of the distal context.

## 1 Introduction

Duration is a common phonetic cue across languages. The perception of word-final voicing in stop consonants in English, for example, is strongly contingent on the duration of the vowel immediately preceding the consonant [1]. Yet duration can also vary according to factors extrinsic to the specific segments in an utterance, including according to variation both between and within talkers that may correspond to individual preference or differences between speech communities in how quickly sentences are spoken [2–4]. Some talkers are faster than others, and some contexts demand fast speech–say, auctions, or emergencies. This variation in speech rate requires listeners to adapt their interpretation of the speech signal [5]. Listeners must change their expectations about, for example, the expected duration of individual segments based on the input that they are getting from the speaker and the context. This phenomenon has been studied in languages such as English [6], Dutch [5], and Maltese [7], generally by asking listeners either to write down sentences that they hear or to make a forced-choice judgment between two possible options This process of adaptation becomes even more interesting when duration and duration alone is the primary cue to a segmental contrast. This paper explores perceptual rate adaptation in a language not yet targeted for studies of the phenomenon: Modern Standard Arabic (MSA). Although duration is almost always one of many cues to the identity of segments in English, in Arabic (and in many other languages), duration itself can help distinguish between singleton and geminate consonants.

MSA contrasts both consonants and vowels in terms of length. That is, words in MSA may be distinguished from one another only in terms of the length (or duration) of individual segments within a word. As an example of the importance of consonant length in Arabic, the words *kabara* [ˈkabaɾa], ‘it became bigger’, and *kabbara* [ˈkabːaɾa], ‘it made [something] bigger’, are differentiated only by the length of the medial [b] sound. (Note: for the present paper, long/geminate consonants will be transcribed using [ː] in the phonetic transcription, making the transcription of ‘it made [something] bigger’ [ˈkabːaɾa]. Short/singleton consonants will lack a [ː]. Transliterations will employ doubled consonants.) These contrasts form an integral part of the grammatical system of Arabic, making them common.

In the present study, we examine the effects of distal speech rate on the perception of geminate consonants in Arabic. What constitutes “distal” information has been inconsistent between and even within experimenters [8, 9], so it is useful to be clear about the definition. In the present paper, by “distal speech rate”, we mean the rate of speech more than a syllable remote from a point of maximal ambiguity within a sentence, following Dilley and Pitt [6] and Heffner et al. [10]. The ambiguous segments being studied are a class of geminates referred to as *heteromorphemic geminates* [11, 12]. In typical typologies of gemination, such as that of Davis [13], they contrast with *monomorphemic geminates*, such as the [bː] in *kabbara*, ‘it made [something] bigger’, which occur organically within a single morpheme (though see Heselwood and Watson [14] for an opposing view). Heteromorphemic geminates, on the other hand, arise from the concatenation of two morphemes, that lead two consonants to be placed next to each other in a way that is realized as a single, long consonant. For example, *al-durūs*, ‘the lesson’ in Arabic is pronounced *ad-durūs* [adːuɾuːs], with a geminate [dː] as the second segment. But this geminate is not inherently specified as a part of the word *durūs*, ‘lessons’; it is traditionally described as arising due to the assimilation of the second segment of the definite clitic *al* with the following consonant [15]. Although this theoretical perspective on geminates in Arabic has been challenged in some cases [14], the details of these arguments do not prevent the conclusion that the apparent gemination present in the signal straddles a morpheme boundary. These two segments are usually pronounced as a single, long consonant [16]. Since they straddle a morpheme boundary, they are referred to collectively as heteromorphemic geminates. This distinction between monomorphemic and heteromorphemic geminates has been studied across a wide variety of phonetic contrasts and languages, including Estonian and American English [17], Turkish and Bengali [18], Levantine Arabic [16], and Tashlhiyt Berber [19]. The present study examines whether heteromorphemic geminates are affected by distal rate adaptation.

### 1.1 Context rate adaptation

Studies of context rate adaptation have come to different conclusions about the strength of rate adaptation effects depending on the target of investigation [6, 8, 20, 21]. For word segmentation, the process of figuring out where one word stops and the next starts in fluent speech, distal rate adaptation effects are generally strong. For lexically ambiguous syllable strings such as *down-town-ship-wreck*, for example, which might be parsed as *downtown shipwreck* or *down township wreck*, the rate of earlier syllables seems to set up expectations about the rate of later syllables. Listeners can go from perceiving *downtown shipwreck* to *down township wreck* based solely on the manipulation of the rate of earlier syllables [22]. EEG investigations indicate that these effects appear as soon as 100ms after a syllable onset [23].

These effects also emerge for function words. Function words, which serve grammatical but not meaning-based purposes in sentences, are contrasted with content words, which bear meaning but do not serve a similar grammatical purpose. Function words are often acoustically reduced in English and other languages [24, 25]; that is, they are produced with a shorter duration and a less distinct articulation than content words. This makes them prime candidates for word boundary ambiguity, as their indistinct nature often makes them blend with their neighbors in terms of their acoustic content.

Many studies of context rate effects in English have examined the extent to which changing the rate in the context of an ambiguous function word can lead to its (perceived) disappearance. For example, within the sentence fragment *Anyone must be a minor or child*, the function word *or* is frequently reduced in prediction; it is pronounced [ɚ] (“er”), matching the pronunciation of the immediately preceding vowel, also [ɚ]. Whether the last words of the fragment are perceived as *minor or child* or *minor child*, then, is contingent on the perception of the length of the [ɚ] sound. Critically, this duration is perceived relative to the rate of the context. Slowing down the first part of the sentence (*Anyone must be a mi-*) can lead to the perceived disappearance of the (ambiguous) function word without any manipulations of the function word itself [6]. In addition to being present for function words, these context rate effects have been shown to be present for the location of word boundaries within consecutive, identical consonants. Heffner et al. [8] found that phrases like *Canadian notes*, with two identical [n] segments straddling a word boundary, could be perceived as *Canadian oats* just by slowing down the rate of the context.

Outside of English, context rate effects on word segmentation have also been shown to be strong. In one study using Dutch [26], for example, listeners heard sentences with phrases ambiguous to the location of a word boundary, such as *eens (s)peer*, ‘once spear/pear’. The pairs were ambiguous to whether two [s] sounds abutted the word boundary or whether there was just a single [s] found to one side of the boundary. Resembling findings from English, the rate of speech more than a syllable remote from the potential word boundary influenced the perception of the word boundary, with a slower distal rate leading people to report the doubled [s] less often. An examination of Russian [27], meanwhile, had a focus more generally on the presence or absence of certain reduced segments within an utterance. This sometimes affected word segmentation, while in other cases the ambiguous segment in question signaled a lexical difference between two words; the difference between *zhdala ya opyat’*, ‘I kept waiting again’, and *zhdala ya pyat’*, ‘I expected a company of five’, was in the presence of the unstressed *o* ([ᴧ]) at the beginning of the last word (*opyat’*). While the distal rate effects varied from item to item and context to context, they were generally of a scale seen in studies of English word segmentation effects; slower distal contexts led to the perception of fewer segments or word boundaries within the region of ambiguity.

These findings within the segmentation literature stand in contrast to studies that have examined the influence of context speech rate on the perception of individual segments. Although it has long been understood that the perception of segments—for example, the perception of the distinction between [b] and [w] [28], between [b] and [p] [28], or between [ʃ] and [tʃ] [29]—can be influenced by the perception of the duration of immediately adjacent segments, support for the idea that segments further away can affect the perception of individual segments is equivocal at best. Distal rate effects from context more than a syllable remote from a point of segmental ambiguity are rare and often weak [30], with some authors proposing that long-distance rate normalization is unnecessary [31] or qualitatively different from the rate adaptation that occurs due to proximal information [20, 21]. Outside of English, Pind [32] found significant but very small effects of rate context on stop voicing perception in Icelandic, resembling in size those uncovered in English.

### 1.2 Geminates

Although many of the examinations of distal rate effects on segmental contrasts show weak evidence for rate adaptation on segmental perception, duration can influence the perception of a wide variety of segmental phenomena. This is seen most clearly in languages that have contrastive consonant length. In many languages, including Italian [33], Kelantan Malay [34], Swiss German [35, 36], Chickasaw [37], Japanese [38], and Arabic (the subject of the present investigation), words can be distinguished merely by the duration of single segments within them. For example, in Arabic, the words *darasa*, ‘he studied’, and *darrasa*, ‘he taught’, are differentiated by the duration of the medial [r]. By contrast, although analogous situations can sometimes arise in English across morpheme boundaries, consonant length alone does not distinguish individual lexical items.

#### 1.2.1 Correlates of gemination in production

In languages that contrast the length of consonants, there are typically two strongly overlapping cues that inform the perception of the consonants: the absolute length of the consonant, and the relative length of the consonant to the previous vowel. Geminate consonants are, unsurprisingly, long in duration when compared to their singleton counterparts [32, 39–41], and listeners use the length of consonants to determine whether they are singletons or geminates [42]. On top of that, consonant length often shows a robust cue-trading relationship with the length of the previous vowel, with longer consonants often being associated with shorter preceding vowels [40, 43]. In Arabic, vowels that precede geminate consonants are relatively short compared to vowels that precede singleton consonants [44, 45]. Yet this particular relationship is subject to some cross-linguistic variability, as seen with Japanese geminate consonants, which are said to be preceded by *longer* vowels than singleton consonants [46], and Polish geminate consonants, which do not seem to trade off with adjacent vowel durations [47].

In languages with segmental length contrasts, variation in duration is not solely contingent on phonetic or phonological factors. In Levantine Arabic, for instance, segments can be lengthened when they are subject to contrastive focus (when a speaker may be pointing out a mishearing, for instance). The length of long vowels tended to change more when found in words under contrastive focus than the length of short vowels [48]. The length of both geminate consonants and the vowels that precede them may also vary according to speech rate, another cue independent of the phonological specification of the consonants themselves.

Thus, listeners face a dilemma resolving this type of input: are segments short because the speaker is talking rapidly, or are they short due to contrastive consonant length? While the length of adjacent syllables can often show variation in line with speech rate, ratios of consonants to vowels have been shown to be relatively stable even in the face of rate variation, at least for Italian and Japanese [49–52]. The authors of these studies have proposed that this reflects an invariant ratio between the length of a consonant and the length of its context that could be used as a reliable cue to perceived consonant length; as a consequence, a consonant with a constant duration might be perceived as either long (i.e., as a geminate) when preceded by a relatively short vowel or as short (i.e., as a singleton) when preceded by a relatively long vowel. In Japanese, at least, the findings seem to implicate a ratio between a geminate consonant and the syllables immediately adjacent to that consonant [53]. In Arabic, singleton consonants tend to be associated with longer preceding vowels, while geminate consonants tend to be associated with shorter preceding vowels [45]. The findings from the production literature outlined above suggest that listeners can show rate adaptation effects (at least in proximal contexts) even in the case of consonant length contrasts that are primarily cued by the same durational properties that signal rate changes.

#### 1.2.2 Correlates of gemination in perception

Despite the evidence for relative cue weighting in production, there is mixed evidence for the use of relative duration in the perception of consonant length. One early study of consonant duration in Turkish and Bengali found no evidence for the use of relative duration on the perception of consonant length [54]. Yet this study has been contradicted by evidence that speakers of Japanese [53] and Persian [55] do seem to rely on ratios of duration. One explanation for these differences in results has to do with variation. At least for Japanese speakers, there is a great deal of variability across individuals in their use of absolute and relative duration in the perception of consonant length contrasts [56]. There is also cross-linguistic variation in the importance of these cues; in one cross-linguistic study of gemination, Japanese speakers exploited relative rate in a way that Finnish speakers did not [57]. Finally, the differences in findings between the studies might be explained by representational properties of geminates. In later studies of Bengali geminates, Roberts et al. [58] and Kotzor et al. [59] found evidence for asymmetrical activation between geminates and singletons, with geminate pronunciations activating singleton words but singleton pronunciations not priming geminate words. The authors ascribed this asymmetry to the phonological representations of singleton and geminate consonants.

A small-sized study of rate adaptation in the perception of Japanese segments indicated that the boundary between short and long consonants (in this case, between [ise] and [isːe]) was strongly dependent on the rate of the context, which consisted of a three-syllable word immediately preceding the ambiguous words and a two-syllable word immediately following the ambiguous words. Slow contexts required longer segment durations for a consonant to be heard as a geminate [60]. Japanese speakers benefit strongly from having previous phonetic information in determining whether an ambiguous segment is long or short. Excising ambiguous words from their sentential contexts, or swapping out one segmental context for another produced at a different rate, led the identification of long or short consonants to be much less accurate [61]. These results have been extended to show that distal speech rate can also affect the perception of geminate consonants in Japanese, regardless of whether the distal speech was produced by the same talker as or a different talker from the talker who produced perceptually ambiguous consonants [62]. Outside of Japanese, in the same study where he examined context duration effects on voicing, Pind [32] showed that listeners were strongly influenced by the length of whole syllables or words when distinguishing between short and long consonants in Icelandic, not just adjacent vowels, indicating possible influence of more than just an adjacent vowel.

Although there are still not many studies of distal context effects on consonant length in other languages, studies of distal context effects on vowel length are more common [5, 63–65]. In some languages, vowels, like consonants, can lead to meaning differences based on their length. These length differences can in turn be affected by the rate of the distal context around a point of ambiguity. Dutch, for instance, contrasts the vowel categories /ɑ/ (as in *mat*, ‘mat’) and /aː/ (as in *maat*, ‘size’) based in large part on the duration of the vowel. These durational differences in turn can be affected by the distal context rate around ambiguous tokens of these vowel categories. Slowing down the distal context led segments from being heard as /aː/ to /ɑ/ [5, 65]. Using eye-tracking data, it has been argued that these effects occur in real time even in the face of countervailing morphosyntactic cues [64]. Even the common perception of a foreign language as being “fast” seems to influence the perception of individual segments as being short or long. Not only do German speakers adapt to the context rate when distinguishing between /a/ and /aː/ in their own native language, but their responses to Dutch sentences are shifted in a way that resembles how they respond to fast sentences in their own native languages [63]. The pattern was less clear for Dutch-speaking participants in the same study, however.

The clearest evidence for the importance of distal speech rate on consonant length perception, and the most relevant paper to the present study, focused on the perception of geminates in Maltese. Maltese is a Semitic language, most closely related to the varieties of Arabic spoken in the western part of North Africa. Like Arabic, it uses contrasts between singleton and geminate consonants to signal important lexical contrasts. Mitterer [7] examined the influence of rate on the distinction between singleton and geminate consonants on Maltese. Unlike previous studies on the perception of geminate consonants, he examined the influence of distal rate—which he defined as the context more than one syllable remote from the ambiguous consonant in particular—on the distinction between singleton and geminate consonants. Using a small set of original items, he created a set of stimuli with the duration of ambiguous consonants set at five different levels and the duration of the distal context set to two levels. He found that listeners were influenced by the rate of the distal context in their assignment of tokens to either long or short values. For the most ambiguous items, the extent to which listeners heard a geminate dropped from nearly 100% (when found in a fast sentence context) to about 50% (when found in a slow sentence context). This provides strong evidence that distal rate can influence the perception of monomorphemic geminates. Neither these results, nor any methods that have involved directly manipulating context speech rate and examining how those manipulations affect the perception of later-occurring information, have been extended to Modern Standard Arabic.

#### 1.2.3 Heteromorphemic geminates

But what of heteromorphemic geminates? Although only some languages have monomorphemic geminates, many languages include contrasts that resemble heteromorphemic geminates. In English, for example, words like *unnamed* and *immoral* are said to have heteromorphemic geminates, caused by the concatenation of the affixes *un-* and *in-* to words beginning with a nasal [66]. In both cases, the words in question have immediately adjacent, identically specified nasals. Indeed, an example of a heteromorphemic geminate was given earlier in this paper: the phrase *Canadian notes* used by Heffner et al. [8] has two adjacent, identical consonants that span a morpheme (and, in this case, a word) boundary. It is not clear whether these heteromorphemic geminates in English and in German have consistent acoustic differences from their singleton peers; although they are certainly longer in many cases, it is not clear if these differences are gradient or discrete, and whether some of them can be explained through the relative ratio of those consonants to the vowels immediately preceding them [11, 66–68]. Even within a single study of Hungarian geminates, the extent to which monomorphemic geminates and heteromorphemic geminates differ seemed to depend on the contrasts being studied [69]. Although studies of perception are rarer, a priming study in Italian showed that Italian speakers are capable of distinguishing monomorphemic and heteromorphemic geminates that could straddle a word boundary [12], making a possible case that the differences that are present can be used by listeners in comprehension.

### 1.3 Current study

The phonetic and phonological realities of Arabic make the language an interesting case study. Arabic consonant length contrasts are also associated with cue trading relationships that resemble those in Italian and other languages [45]. Thus, listeners must decide whether any segment has a short duration due to a meaningful contrast in the language or due to speech rate, making it important to adjust to speech rate to accurately perceive those meaningful contrasts.

The present study describes an experiment investigating distal rate adaptation effects on Arabic speakers’ perception of heteromorphemic geminates. We seek to replicate the findings of researchers who have examined Maltese [7], English [6], Dutch [64], and Russian [27] using MSA and a relatively diverse stimulus set. This is, to our knowledge, the first study in Modern Standard Arabic examining the influence of distal speech rate context on Arabic geminate perception, making it essential to determine the cross-linguistic validity of a set of results heretofore largely studied (apart from Maltese) in Indo-European languages. Within the domain of geminates, we aim to extend the previous findings for monomorphemic geminates to heteromorphemic geminates, which straddle two separate morphemes. We hypothesize that slowing down the distal speech rate around a consonant that does not have syntactic or semantic context that would lead it to be processed as a singleton and geminate should make that consonant sound more like a singleton while speeding up the distal speech rate should make that consonant sound more like a geminate. This is in line with the idea that consonant duration is perceived relative to the context. To our knowledge, this is not only the first study examining heteromorphemic geminates in a Semitic language, but moreover the first study heteromorphemic geminates across a clitic boundary or involving morphophonemic processes.

## 2 Methods

### 2.1 Participants

20 people participated in this experiment (16 female, 3 male, 1 not stated). All were at least 18 years old (*M* = 27.9, Range = 19–50) and had no history of speech or hearing disorders. Not all participants were comfortable giving their exact range and instead gave an age range; the midpoint of that range was used for the calculation of the mean and range. All were native speakers of Arabic, primarily Peninsular Arabic, and were fluent speakers of Modern Standard Arabic, the standardized variety of Arabic used in writing and in mass media in the Arabic-speaking world. Participants were recruited either in the United States or Saudi Arabia by a native speaker of Arabic. They were compensated at either a $10/hour wage or local equivalent or refused payment. The experiment was performed in line with the guidelines of the University of Maryland, College Park Institutional Review Board (IRB), which included oral consent. Datasets were collected between August 11, 2015, and May 28, 2016.

### 2.2 Materials

In the present experiment, 30 sentence pairs were designed with a critical ambiguity in the length of a consonant signaling the presence of a definite clitic. In Arabic, the definite clitic is often transliterated as *al*, and is attached to the beginning of a noun or an adjective that it modifies; *bayt*, ‘a house’, becomes *al-bayt*, ‘the house’. Two key processes can conspire to render its perception dependent only on the length of a critical consonant. First, when the definite clitic is attached to a noun starting with a coronal consonant, the assimilation of the /l/ to the following coronal ensures that the consonant is pronounced as a geminate rather than as a singleton. For example, the definite form of the noun *sayarāt*, ‘cars’, *al-sayarāt*, is pronounced *as-sayarāt* [asːajaɾaːt], ‘the cars’. This makes the length of the consonant phonetically long ([sː]); it is, thus, a heteromorphemic geminate, as it straddles a morpheme boundary [16]. Second, when articulated after a word ending with a vowel, the *a* of *al* is usually elided in speech. An example of this is in the phrase *baʿḍu al-sayarāt*, ‘some of the cars’. Despite the presence of the *a* in the transliteration, when the word *baʿḍu* ([baʕdˁu]), ‘some’, precedes *al-sayarāt*, ‘the car’, the *a* of *al-* [as] is often elided in pronunciation yielding [baʕdˁusːajaraːt]. In cases when the determiner clitic is preceded by a vowel-final word and attaches to a word starting with a coronal consonant, the only disambiguating phonetic cue as to whether the clitic is present is whether the coronal consonant is short (if absent) or long (if present).

**Fig 1:**
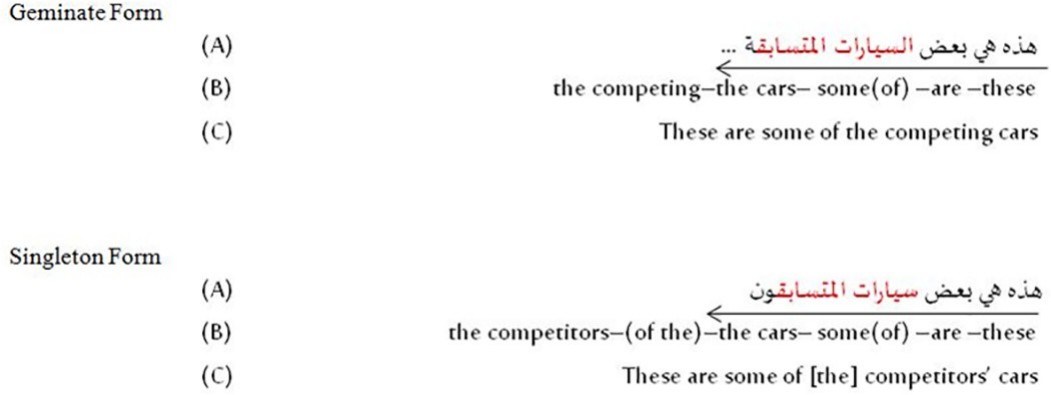
Experimental stimulus example.

The sentence pairs that were created for this experiment differed only in the length of a consonant that signaled the presence of the definite clitic and, thus, the presence of a heteromorphemic geminate. To do this, 30 sentences were constructed that were identical up to the point of a critical consonant, then diverged with regard to the length of that consonant. The items that were recorded without an *al* will be referred to as “singleton” items, while the items with an *al* will be referred to as “geminate” items (see Fig 1 for examples of pairs). The first line for each item shows the stimulus sentence in Arabic script, the second line in a Romanization of the orthography with morpheme boundaries marked, and the third line in a broad transcription of the sentence’s intended pronunciation, or the portion of it that participants heard. The fourth and fifth lines provide a morpheme-by-morpheme gloss and an idiomatic English translation of the Arabic sentence.

Although later-occurring grammatical and semantic information that disambiguated the length of the critical consonant was included to aid in the speakers’ pronunciation of the materials, this information—the final syllable in the two sentences given as examples—was cut out of the sentence fragments that were played for the participants. 60 filler sentences with no such ambiguities were also constructed, with those sentences being subject to truncation in a similar way to the experimental items. Four native Arabic speakers—two female speakers of Peninsular Najdi Arabic, and two male speakers of Egyptian Arabic—recorded the stimuli using MSA in a sound-attenuated booth using a Shure SM51 (Niles, Illinois, USA) microphone attached to a Scarlett Focusrite 2i2 Audio Interface (High Wycombe, England, UK) at a sampling rate of 44100 Hz. The voice talents were given time to review the materials, ask questions, and perform a sample recording. Once the sample audios were checked for clarity, the voice talents were asked to continue recording at their own pace. Voice talents were also encouraged to re-record items if an error was made. The final set of recorded items by each voice talent was checked again to ensure that the items were read clearly and correctly. Items were selected to roughly balance the number of items used from each of the four speakers while also selecting singleton and geminate items that had similar acoustic properties. Although varieties of spoken MSA in Saudi Arabia and Egypt do have some phonetic and phonological differences, all varieties of Arabic commonly spoken in either of these countries include the phonological properties that ensure the primary cue to the presence of the definite article is the duration of a critical consonant, including vowel elision and complete coronal assimilation.

The 30 items with the critical ambiguity had their distal speech rates modified. The distal context was defined as anything more than a syllable remote from the point of ambiguity, in line with previous studies of these effects in word segmentation [6, 7, 10]. The proximal context was defined as everything within a single syllable of the word boundary. For these items, there were three possible context rates: Normal (with no change to the distal rate), Slow (with a distal context length set to 175% of the unmodified version), and Fast (with a distal context length set to 70% of the original duration). The rate of the filler items was also changed to be Normal, Slow, or Fast, but with rate manipulations that affected entire sentences rather than parts of the sentences. All items were also intensity-normalized to an average intensity of 70 dB SPL.

### 2.3 Procedure

The experiment used a 2 (Type: Singleton or Geminate) × 3 (Distal Rate: Normal, Slow, or Fast) design. Items were randomly assigned to one of six lists, where each experimental item was assigned to one of the six combinations of Type and Distal Rate. For half of the lists, one item was inadvertently added to the list twice; the second iteration of that item was removed from further analysis. The order of all experimental and filler items was completely randomized for every participant, except for two filler items used for practice at the beginning of the experiment. All participants completed the study using the same computer and headphones and were encouraged to ask questions before they started the experiment. Participants were informed that the experiment in its entirety, from introducing the study to getting consent to the debrief at the end of the session, would take no more than 80 minutes.

PsychoPy [70] was used to run the items, and participants heard the sentences presented one-by-one and were asked to write down the sentences that they had heard. To ensure that participants’ transcriptions reflected accurate content words, participants could repeat an item up to five times before they wrote the sentence down, a number chosen based on piloting. Specifically, participants were informed that they would hear a sentence in Arabic that would be repeated automatically five times. However, they could press the space bar to begin transcription if they wished. Once they heard the sentence, participants were instructed to write it down in standard Arabic script on a provided notebook to the best of their abilities. They were told not to worry about spelling, punctuation, or writing style, as the goal was to simply write what they had heard. Once they wrote down the sentence, they moved on to the next item by pressing the space bar. Thus, participants proceeded through the experiment at their own pace; they were allowed to determine when they started transcription and then they moved on to the next trial. Participants were pseudorandomly assigned to one of six counterbalanced lists, with each experimental item for each participant being played at a single combination of type and distal rate, and each filler being played at a single rate. Excluding the practice session, each participant thus heard a total of 90 sentences (30 experimental, 60 filler). Following the completion of the experiment, participants partook in a brief demographic and exit questionnaire and engaged in a debrief session with the researcher. Most participants completed the study within an hour to an hour and twenty minutes.

### 2.4 Analysis

The dependent variable was the trial-by-trial presence of the critical determiner clitic *al* in participants’ transcriptions, coded in a binary fashion. To assess this, participants’ transcriptions of each sentence were examined. For a few trials (less than 5%), it was indeterminate whether the participant had transcribed the determiner given transcription errors near the critical region. These trials were not considered for subsequent analysis. However, for the rest, the presence of the definite clitic was coded as either a 1 if the transcription contained the determiner or a 0 if the transcription did not.

The fixed factors were stimulus type and distal rate, the creation of which was described in the “materials” section. Type was coded as a categorical factor with two levels: geminate and singleton. Distal rate, meanwhile, was coded as a continuous factor to preserve the fact that the three rates were not categorical; rate exists along a continuum. The factor levels expressing the duration of the distal duration with regard to the unmodified version of the clip were base-2 logarithmically scaled to give the numbers that each rate was coded as: the Normal rate was coded as 0, the Slow rate as 0.807 (the base-2 logarithm of 1.75), and Fast as -0.515 (the base-2 logarithm of 0.70). Logarithmic scaling reflects the fact that the underlying values used (1.75 and 0.70) are ratios relative to the originally recorded files; logarithmic scaling is more appropriate for ratio-based measures.

Binomial generalized linear mixed-effects models were then implemented in the lme4 package [71] to compare participants’ tendencies to transcribe the definite clitic across combinations of Type and Distal Rate. The approach we adopted involved model comparison procedures. Model comparison procedures are meant to balance considerations of completeness (adding every possible random or fixed factor) and parsimony (adding no additional predictors beyond what is necessary) when determining the optimal model to fit a dataset. Effectively, the procedure employed here compares more fully specified models with simpler models that lack a predictor using chi-squared tests of model fit. The chi-squared tests depend mathematically on the deviance of the models being compared, a value that depends both on the complexity of the model and the goodness of fit of the model. If the chi-squared tests indicate that there is a significant difference between the models in fit, the predictor contributes significantly to the final model. If there is no significant difference, the predictor can be safely removed from the final model, because the inclusion of that predictor does not explain sufficient variance to overcome the introduction of additional complexity in the model. This process is repeated until the final relevant model, the simplest one to explain the variation in the dataset, is chosen. The use of model comparison is common in speech perception studies [8, 72–78].

A model comparison procedure was used to first identify the most complex random effects structure supported by the data, with help from procedures instantiated within the RePsychLing package [79]. The range of random factors that were considered included random intercepts for participant (to allow participants to vary in their likelihood of perceiving the definite clitic) and for item (to allow items to vary in the likelihood that they are associated with a definite clitic), and random slopes for each fixed factor by participant and item (to allow participants and items to vary randomly in the extent to which the fixed factors influence them). Model comparison was next used to determine the fixed effects and interactions with a significant impact on participants’ transcriptions of the critical region [80]. The fixed effects considered included distal rate, type, and the interaction between them.

## 3 Results

**Fig 2:**
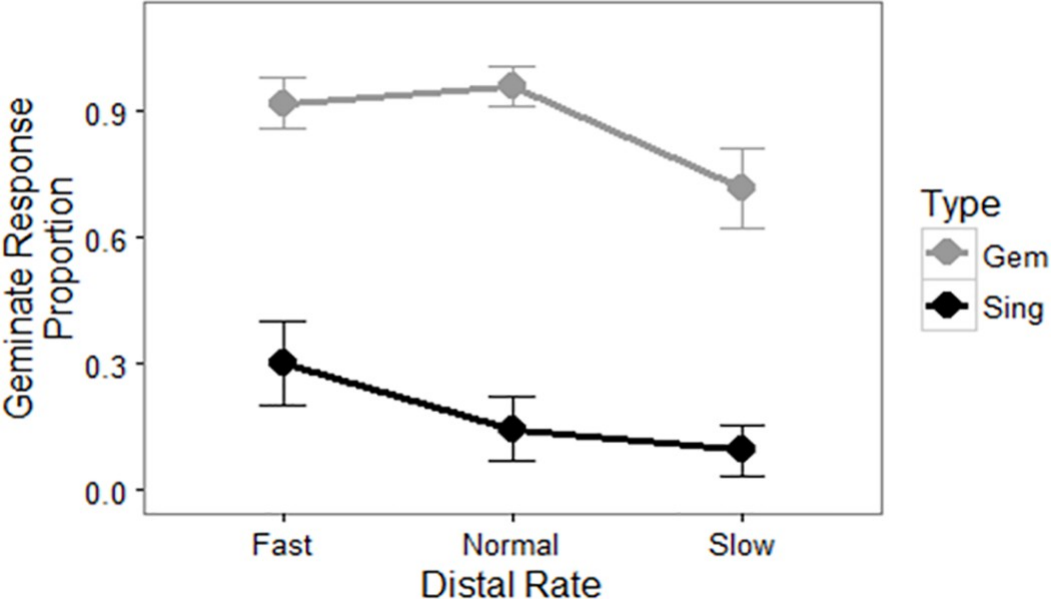
Results for experimental items, showing the proportion of critical regions transcribed with a geminate response, by original item type (geminate or singleton, also indicated with shading) and distal rate (fast, normal, or slow). Error bars are by-participant standard errors.

A summary of the results in the experiment is found in Fig 2. The item type (singular versus geminate) clearly affected the proportion of trials in which participants reported a geminate consonant. However, the slope of each line also indicated support for the idea of distal rate effects on the perception of the critical consonants as well.

These subjective observations were confirmed using model comparison in the context of generalized linear mixed-effects modeling. The first step was to assess the ideal random effects structure for the dataset. To do this, an initial model was constructed that had all the potential fixed and random effects included. No correlation parameters were included between random slopes [80]. To find the maximum number of dimensions supported by the random variation in the data, a principal components analysis (PCA) was performed on the variance-covariance matrix of the model using the RePsychLing package in R [79] to put an upper limit on the number of random effects considered. This PCA indicated that a maximum of three random components were supported by item, but only one was supported by participant. Removing the random slopes by participant showed no significant change in model fit, *χ*^2^(2) = 3.18, *p* = .20. Thus, the intermediate model, with random intercepts by participant and by item and random slopes for distal rate and type by item, appears to provide the most reliable set of random effects.

This intermediate model was then compared to models that lack different fixed effects to determine the significance of each of these effects. There was a significant decrease in model fit with the fixed effects of type removed, *χ*^2^(2) = 66.0, *p* < .001. Comparing the intermediate model to one lacking distal rate also yielded a significant decrease in model fit, *χ*^2^(2) = 15.4, *p* < .001. However, comparing the intermediate model to a final model lacking solely the interaction term showed no significant change in model fit without the interaction, *χ*^2^(1) = 0.267, *p* = .61. This suggests that including the interaction between distal rate and type in the model is not parsimonious; any additional variance explained by the fixed interaction between those terms was more than offset by the additional complexity of the full model.

The dataset, then, best supports a final model that includes distal rate and type as independent fixed factors. Fixed model parameters are available in Table 1, with Geminate as the reference level for the type factor. The fixed effect of type indicates that participants were much more likely to hear the critical consonant as a geminate if it was recorded with that intention, probably in line with the many other acoustic cues present that can indicate the presence of a geminate consonant [39, 40, 57]. The fixed effect of distal rate indicates that slowing the distal rate made participants less likely to hear the critical consonant as a geminate.

**Table 1:** The best-fitting model in the present experiment.

Factor	Estimate (*b*)	*z*	*p*
Intercept	2.62	8.01	< .001
Distal Rate	-1.90	-3.59	< .001
Type:Singleton	-4.48	-10.6	< .001

## 4 General discussion

The experiment described in this paper tested the effects of distal rate adaptation on the perception of Modern Standard Arabic (MSA) geminate length contrasts. Specifically, we examined the perception of heteromorphemic geminates, which straddled the boundary between various nouns and the definite clitic that could be attached to them. Based on the literature from English and from monomorphemic geminates in Maltese [7], we predicted that speeding up the distal context around a singleton consonant in Arabic would increase the likelihood for that consonant to be perceived as a geminate. Slowing down the distal context around a geminate consonant, on the other hand, would increase the likelihood for that consonant to be perceived as a singleton. The findings of the study supported the predictions completely. Fast distal contexts led singleton consonants to sound relatively long, and thus increasing the chance that they were perceived as geminates; slow distal contexts led geminate consonants to sound relatively short, and thus had an increased chance of being perceived as singletons. This suggests that, when it comes to distal rate adaptation, the phonetic correlates of gemination pattern with the phonetic correlates of word segmentation that are dependent on the presence of consonants or reduced vowels located near word boundaries [6, 8, 26, 27] rather than the correlates of phonological features such as voicing or affrication [8, 20, 30, 32]. This replicates and extends the previous literature for word segmentation in English and monomorphemic geminates in Maltese to a novel morphological setting: geminates that appear as a result of a morphophonological process, arising from the attachment of a clitic to a content word.

The patterns observed in the results of these experiments more closely match the literature on distal rate adaptation effects for word segmentation contrasts, not for segmental contrasts involving features such as voicing. Examples of segmentation-based contrasts in English include pairs such as *Minneapolis sale* and *Minneapolis ale*. Distinguishing between the two possible ways to segment the phrase depends critically on the perception of the length of the ambiguous [s] sound. If the sound is long enough to be perceived as two instances of [s], the phrase is perceived as *Minneapolis sale*; if not, it is perceived as *Minneapolis ale*. In each of these instances, context rate appears to strongly influence the perception of the length of this class of consonants [8, 26, 81]. This can be compared to studies of distal rate adaptation on the contrasts that are meant to distinguish the identity of a segment—including, say, contrasts between affricates and fricatives, or voiced and voiceless consonants—where adaptation effects are said to be very small or even non-existent [20, 21]. What could explain the difference between the studies of segmental perception and segmentation? Heffner et al. [8] studied this question in American English, comparing closely-matched sentences that differed in either the location of a word boundary (*Canadian oats* vs. *Canadian notes*) or the voicing of a boundary-adjacent consonant (*Canadian coats* vs. *Canadian goats*). Four possible explanations were considered.

Two of these relied on a qualitative split between segments and segmentation, either in terms of how information is processed or in terms of how information is represented. The two other explanations related to idiosyncratic differences between the previous studies in the literature, either in terms of what was considered to be “distal” in discussions of rate context or in terms of the types of items that were used in the previous studies. Here, we add heteromorphemic geminates to the list of contrasts that are modulated by distal context rate. The findings in the present study provide more support for the idea of a qualitative split between segment identity and segmentation.

There is one interesting counterpoint to this suggestion, however. A majority of the studies that fail to show distal rate adaptation effects are ones that have involved initial voicing contrasts. This might occur because voice onset times (VOTs) are perhaps not as rate-dependent as originally thought; the VOTs alone, without recourse to the rate of the surrounding syllables, may provide sufficient information to distinguish between voiced and voiceless tokens [31]. However, the same was not true for word-final voicing perception, where context rate did have an effect on whether a voiced or voiceless word-final segment was perceived [8]. Thus, there is some evidence that aspects of segments such as voicing can be represented in a way that connects with aspects of timing. Word-initial stop consonants may resist context rate effects as a form a cue trading. If VOT strongly predicts word-initial voicing, listeners may ignore other cues to it like distal speech rate. Listeners in more uncertain conditions, such as with word-final voicing or gemination, may instead rely on a broader set of less consistent cues.

More generally, this work reinforces the ubiquity of distal context rate effects in speech perception. One useful future extension of this project is to examine the rate adaptation of L2 learners. If the effects seen here are truly one and the same as the effects of distal rate on word segmentation in English, then English-speaking L2 learners of Arabic should also show rate adaptation effects early in acquisition. Indeed, although English speakers sometimes misattribute consonant length as reflecting aspects of the pronunciation of adjacent vowels [82], other studies have found that English speakers without training can sometimes identify [83] or discriminate between [46] short and long consonants in a way resembling the abilities of native speakers of languages that have (homomorphemic) geminates. An examination of the evolution of rate effects over the course of acquisition, similar to previous studies of production, identification, and discrimination [84–86], could shed light on the universality of rate context effects in perception.

## 5 Conclusions

In sum, this paper replicated and extended findings from English [6], Russian [27], Dutch [5], and Maltese [7] indicating that speech rate context can affect the perception of speech perceptual contrasts in Modern Standard Arabic. This suggests that heteromorphemic geminates can be affected by the rate of speech of preceding sentence context. Previous studies examined changes in the perceived identity [5, 27] or presence [6, 27] of full words, or to the conjugation of a verb [7]; the present one also adds clitics to the list of linguistic units that may be altered by distal speech rate. This puts English word-initial stop voicing contrasts, which show only weak context effects [20, 21], in an increasingly anomalous position, as the current study provides additional evidence of another contrast that is strongly sensitive to context rate. Future studies could explore second-language learners or homomorphemic contrasts in Arabic.
